# Modulation of Calcium Oxalate Crystal Growth and Protection from Oxidatively Damaged Renal Epithelial Cells of Corn Silk Polysaccharides with Different Molecular Weights

**DOI:** 10.1155/2020/6982948

**Published:** 2020-01-22

**Authors:** Jia-Yun Chen, Xin-Yuan Sun, Jian-Ming Ouyang

**Affiliations:** ^1^Department of Chemistry, Jinan University, Guangzhou 510632, China; ^2^Institute of Biomineralization and Lithiasis Research, Jinan University, Guangzhou 510632, China; ^3^Department of Urology, Minimally Invasive Surgery Center, The First Affiliated Hospital of Guangzhou Medical University, Guangdong Key Laboratory of Urology, Guangzhou Institute of Urology, China

## Abstract

Corn silk polysaccharide (CSP0; molecular weight = 124 kDa) was degraded by ultrasonication to obtain five degraded polysaccharides, namely, CSP1, CSP2, CSP3, CSP4, and CSP5, with molecular weights of 26.1, 12.2, 6.0, 3.5, and 2.0 kDa, respectively. The structures of these polysaccharides were characterized by FT-IR, ^1^H NMR, and ^13^C NMR analyses. The antioxidant activities, including scavenging ability for hydroxyl radicals and DPPH free radicals, chelation ability for Fe^2+^ ions, and reducing ability of CSP increased with decreased molecular weight of CSPs within 6.0 to 124 kDa. However, antioxidant activity weakened when the molecular weight of CSPs reached 3.5 and 2 kDa. CSP3 with a molecular weight of 6.0 kDa exhibited the strongest antioxidant activity. After protection with 60 *μ*g/mL CSPs, the viability of human renal proximal tubular epithelial cells (HK-2) damaged by nano-COM crystals increased, the level of reactive oxygen species decreased, and the amount of COM crystal adhered onto the cell surface decreased. The ability of CSPs to protect cells from CaOx crystal damage was consistent with their antioxidant activity. CSPs can specifically combine with CaOx crystal to inhibit the conversion of calcium oxalate dihydrate crystal to calcium oxalate monohydrate crystal. All these results showed that the activity of CSPs was closely correlated with molecular weight. A very high or low molecular weight of CSPs was not conducive to their activity. CSPs, especially CSP3 with a molecular weight of 6.0 kDa, can be used as a potential antistone drug.

## 1. Introduction

Kidney stone is a common disease that affects 10% to 15% of the population and has a high recurrence rate [1]. At present, no effective method has been developed to prevent kidney stone formation and delay its recurrence. The components of kidney stones are primarily calcium oxalate (CaOx), including calcium oxalate monohydrate (COM) and calcium oxalate dihydrate (COD) [2].

The formation of kidney stones is closely correlated to the heterogeneous nucleation, growth, and aggregation of minerals and the adhesion of crystals onto renal epithelial cells caused by urine supersaturation [3]. Renal epithelial cell injury aggravates the risk of kidney stone formation [4]. However, when the damaged renal epithelial cells are repaired [5] or the cells are preprotected [6], the probability of kidney stone formation can be greatly reduced. Exogenous plant polysaccharides with antioxidant function can effectively preprotect cells [7] and improve the ability of cells to resist oxidative damage.

Plant polysaccharides can also regulate the growth of CaOx crystals and inhibit the adhesion of crystals onto renal epithelial cells [6]. Gomes et al. [8] investigated the antiurolithic ability of sulfated seaweed polysaccharide (SP) and found that SP can change the surface charge of crystal, induce the morphology of CaOx crystal to become round and blunt, and reduce the crystal size. Huang et al. [9] explored the effects of five kinds of plant polysaccharides with different carboxyl contents on the growth of CaOx crystals. The results showed that five kinds of plant polysaccharides can inhibit the growth of COM, induce the formation of COD, and reduce the aggregation of crystals. At the same time, polysaccharides with higher carboxyl contents showed excellent crystal control ability.

Plant polysaccharides have biocompatibility, biodegradability, nontoxicity, and some specific therapeutic activities [10, 11]. The biological activity of a polysaccharide primarily depends on its molecular weight, and type and content of acidic groups [12].

Corn silk is considered as a traditional natural medicine and functional food in the United States, China, Turkey, France, and other countries. Corn silk has antioxidant, antidiabetic, and antitumor activities [13, 14]. Corn silk polysaccharide (CSP) is the most important component of corn silk [12]. Guo et al. [15] found that CSP has the ability to scavenge DPPH and hydroxyl radicals and can protect L6 skeletal muscle cells from H_2_O_2_-induced oxidative damage. Zhao et al. [16] showed that no symptoms, such as mortality and toxicity, were observed in CSP-treated mice, and CSP can significantly prolong the antifatigue time of mice.

Natural CSPs have difficulty crossing cell membrane and entering cells to play a role because of their large molecular weight [5, 17, 18]. Degradation of CSPs with large molecular weight can better exert their biological activities. The ultrasonic method is used to degrade CSP. In this method, because no reagent is added, the degraded product is easier to purify and has the advantages of environmental protection, convenience, and high efficiency [19, 20].

Hence, natural corn silk polysaccharide (CSP0) with molecular weight of 124 kDa was degraded by ultrasonic wave, and five degraded polysaccharides with molecular weight of 2.0 to 26.1 kDa were obtained. The antioxidant activity, protective ability to HK-2 cells, and regulation ability to CaOx crystals of each CSP were compared and examined, hoping to provide experimental basis for further exploring the formation mechanism of kidney stones and developing new antistone drugs.

## 2. Materials and Methods

### 2.1. Materials and Apparatus

Corn silk polysaccharide (CSP0) was produced by Shaanxi Ciyuan Biological Company. Human kidney proximal tubular epithelial (HK-2) cells were purchased from Shanghai Cell Bank, Chinese Academy of Sciences (Shanghai, China). Calcium oxalate monohydrate (COM) with a size of about 100 nm was synthesized according to the previous reference [21].

Fetal bovine serum and DMEM-F12 culture medium were purchased from HyClone Biochemical Products Co. Ltd. (Beijing, China). Cell proliferation assay kit (CCK-8) was purchased from Dojindo Laboratory (Kumamoto, Japan). Reactive oxygen detection kit (DCFH-DA) and rabbit anti-rat (FITC-IgG) were all purchased from Shanghai Beyotime Bio-Tech Co., Ltd. (Shanghai, China). Phosphate-buffered solution (PBS), phenazine, 1,1-diphenyl-2-picrylhydrazyl (DPPH), phenanthroline and other conventional reagents were of analytical grade and were purchased from Guangzhou Chemical Reagent Company (Guangzhou, China).

Apparatuses that were used are as follows: Fourier-transform infrared spectrometer (FT-IR, Nicolet, American), nuclear magnetic resonance (Varian Bruker-500 MHz, Bruker, Germangy), ultrasonic apparatus (aligned with JP-100S), Ubbelohde capillary viscometer (0.4–0.5, Qihang Glass Instrument Factory, Shanghai, China), thermogravimetric analyzer (TGA/DSC 3+, Mettler Toledo, USA), the apparatus included D/max 2400 X-ray powder diffractometer (Rigaku, Japan), field emission scanning electron microscope (ULTRA55, Zeiss, Germany), and OPTIMA-2000DV inductively coupled plasma (ICP) (ICP-AES, Optima 2000DV, PerkinElmer, CT, USA, Zetasizer nano ZS90, fluorescence microscope (IX51, OLYMPUS, Japan), flow cytometry (FACS Aria, BD Company, USA), and enzyme mark instrument (SafireZ, Tecan, Switzerland).

### 2.2. Degradation of CSP and Characterization

1. *Degradation*. Using ultrasonic technology, CSP0 with a molecular weight of 124 kDa was degraded at room temperature for different time (Table 1). CSP0 was dissolved in distilled water at 5 mg/mL and ultrasonic frequency was 600 W, 40 kHz. After ultrasonic treatment, the degraded solution was then concentrated to one-third of its original volume at 60°C. The product was precipitated by three times addition of anhydrous ethanol [19]. The solution was stored overnight and then filtered, then washed with anhydrous ethanol twice. The degraded polysaccharide (CSPs) was obtained by drying in vacuum.
2. *Measurement of Molecular Weights of CSPs*. According to the literature [17], the molecular weight of polysaccharide was determined by Ubbelohde viscosity method. The relationship between intrinsic viscosity [*η*] and the molecular weight *M* of CSPs could be described by the Mark–Houwink empirical equation *η* = *κM*^*α*^. For CSP: *κ* = 0.0288, *α* = 0.5 [12, 17]. The final value is the average of three parallel experiments.
3. *Determination of Carboxyl (-COOH) Content in CSPs*. COOH content of CSPs was determined by the method of conductometric titration [22]. Each experiment was repeated three times and averaged.
4. *FT-IR Characterization of CSPs*. FT-IR spectra of polysaccharides were determined using films prepared by 2.0 mg of dry CSP sample and 200 mg KBr in the wavenumber range of 4000-400 cm^−1^.
5. *^1^H and ^13^C NMR Characterization of CSPs*. According to reference [23], approximately 20 mg of dry CSP sample was dissolved in 0.5 mL of deuterated water (D_2_O) in NMR tube. After completely dissolved, the polysaccharide sample is put into a magnetic field of a nuclear magnetic resonance spectrometer for detection.

### 2.3. Antioxidant Activity Detection of Corn Silk Polysaccharides

1. *Hydroxyl Radical (·OH) Scavenging Capacity* [24]. The ·OH scavenging ability of polysaccharide in vitro was detected by H_2_O_2_/Fe system method.
2. *DPPH Radical Scavenging Capacity* [25]. 3 mL CSP with different molecular weights (0.15-3 mg/mL) was mixed with DPPH (0.4 mmol/L, 1 mL). The mixture was incubated in the dark at 37°C for 30 min. The absorbance is detected at 517 nm to reflect the DPPH radical scavenging ability of polysaccharide.
3. *Reducing Power* [26]. 2.5 mL CSP with different molecular weight (0.15-3.0 mg/mL) was mixed with 2 mL phosphate buffer (PBS, pH = 6.6) and 2.5 mL 1% K_3_[Fe(CN)_6_]. The mixture was incubated at 50°C for 30 min. 2.5 mL 10% trichloroacetic acid was added to the mixture which was then centrifuged for 10 min at 3000 r/min. The supernatant (2.5 mL) was mixed with 0.5 mL FeCl_3_·6H_2_O (0.1%, *w*/*v*) solution and 5 mL distilled water. The solution was mixed fully and left to stand for 10 min. Then, the absorbance was measured at 700 nm.
4. *Ferrous Ion-Chelating Capacity* [27]. 1 mL CSP with different molecular weights (0.15-3.0 mg/mL) was mixed with 2.25 mL distilled water and 0.05 mL 2.0 mmol/L ferrous chloride solution, respectively, and the reaction lasts for 30 s. Next, the solutions were mixed with 0.2 mL phenazine (5.0 mmol/L) and reacted at room temperature for 10 min, and the absorbance of the mixture was measured at 562 nm. EDTA-2Na was used as the positive control group.

### 2.4. Regulation of Calcium Oxalate Crystal Growth by Corn Silk Polysaccharides

(1) *Calcium Oxalate Crystal Synthesis*. CaOx metastable solution was prepared in a 50 mL volumetric flask by adding 3.0 mL of 10 mmol/L CaCl_2_, 1 mL of 0.50 mol/L NaCl, and a final concentration of 0.5 mg/mL CSP. Distilled water was added to about 46 mL and 3.0 mL of 10 mmol/L Na_2_Ox was added. The volume of the solution was diluted to scale with distilled water. The crystal was filtered by 0.22 *μ*m microporous membrane. The solution obtained was c(Ca^2+^) = c(Ox^2−^) = 0.60 mmol/L and c(NaCl) = 10 mmol/L [28, 29]. The CaOx solution was poured into a 50 mL beaker for crystallization, and a clean glass slide was placed at the bottom of the beaker. To prevent supersaturation of the system due to volatilization of solvent water from driving crystal formation, crystal growth was performed under static conditions. After the crystal grew for 3 d, the substrate was removed and dried in a dryer. The concentration of soluble Ca^2+^ ions in the supernatant was measured through inductively coupled plasma (ICP) emission spectrometry.
(2) *Characterization of Crystal Structure*. The abovesynthesized CaOx crystals were characterized by FT-IR, SEM, and X-ray diffraction. Dried CaOx sample (2.0 mg) was mixed with KBr (200 mg), then grinding with agate mortar, tableting, and scanning with infrared spectrometer in the wavenumber range of 4000-400 cm^−1^. The synthesized crystals were analyzed in an X-ray diffractometer under the test conditions of CuK*α* ray, graphite monochromator, 30 kV, 25 mA, scanning range of 5-60°, scanning speed of 8°/min, and step width of 0.02°/s for qualitative and quantitative analysis. The samples were treated with gold spray and observed under a field emission scanning electron microscope for morphology analysis.
 The relative percentage contents of COM and COD in the CaOx precipitates were calculated through the *K* value method and the relative percentage contents of COD:
  (1) $$\begin{array}{c} \text{COD}\%=\frac{I_{\text{COD}}}{I_{\text{COM}}+I_{\text{COD}}}\times 100\%, \end{array}$$
  where *I*_COM_ and *I*_COD_ are the intensities of the spacing ($\overset{¯}{1}01$) of COM and (200) of COD crystals.
(3) *Zeta Potential Determination of Crystal Surface*. CaOx crystals (1 mg) were dispersed in 3.0 mL of double distilled water. After ultrasonication for 10 min, the zeta potential was detected with a Zetasizer Nano ZS90 apparatus at 25°C.
(4) *Thermal Stability Characterization of Crystals*. According to Reference [30], the thermostability of CaOx crystals was investigated by thermogravimetric analysis on Mettler Toledo thermal analyzer under nitrogen atmosphere from 25°C to 900°C at a heating rate 10°C/min.
(5) *Effect of Polysaccharides on Crystallization Kinetics of Calcium Oxalate Crystals*. The solutions were prepared at concentrations of 1 × 10^−1^, 1 × 10^−2^, 1 × 10^−3^, 1 × 10^−4^, and 1 × 10^−5^ mol/L CaCl_2_. The calcium ion-selective electrode was selected as the negative electrode of the pH acidometer. The calomel electrode was used as the positive electrode of the pH acidity meter. Two electrodes were inserted into the above solution, and the potential values (*E*) at different concentrations were read out from diluted to concentrated by the pH acidity meter. *LgC* is plotted on the abscissa (*x*), and *E* is plotted on the ordinate (*y*), and the linear regression equation is obtained.

The prepared CaOx metastable solution was placed in a 50 mL beaker, the concentration of Ca^2+^ in the solution was detected by using an ion-selective electrode, and the inhibition percentage of CSP on CaOx crystallization was calculated according to the inhibition ratio = (1 − *r*_inhibitor_/*r*_control_) × 100% [29].

### 2.5. Cytotoxicity Measurement of Corn Silk Polysaccharides on HK-2 Cells

Because CSP0 has a large molecular weight (124 kDa) and is not easy to enter cells [22], we chose CSP1, CSP2, CSP3, CSP4, and CSP5 for the subsequent cell tests.

Cell suspension with a cell concentration of 1.0 × 10^5^ cells/mL and 100 *μ*L/well was inoculated per well in 96-well plates and incubated in an incubator in a 5% CO_2_ environment at 37°C for 24 h. Cells were confluent into monolayers and incubated in serum-free DMEM medium for 12 h; the culture solution was discarded. And then 0.1 mL of 60 and 100 *μ*g/mL CSPs with various molecular weights were added and each concentration was repeated in five parallel wells. At the same time, the cell control group (polysaccharide concentration: 0 *μ*g/mL) was set up. After incubation for 24 h, the OD value was measured by using the enzyme mark instrument at 450 nm according to the CCK-8 kit instruction.

### 2.6. Polysaccharides Protect HK-2 Cells from Damage by Nano-COM Crystals

1. *Cell Viability Detection by CCK-8*. After cells were confluent into monolayer, the experiment was divided into 3 groups: (1) the normal control group: in which only serum-free culture medium was added; (2) the damaged group: in which serum-free culture medium with 200 *μ*g/mL COM was added and incubated for 12 h; and (3) the protection group: CSPs with different molecular weights of 20, 40, 60, 80, and 100 *μ*g/mL were mixed with 200 *μ*g/mL COM for 15 min and then cocultured with cells for 12 h. Five multiple wells were set up for each experiment. The OD value was measured by using the enzyme mark instrument at 450 nm according to the CCK-8 kit instruction.
2. *Reactive Oxygen Species (ROS) Level Detection*. 1.0 mL of cells suspension with a concentration of 1.0 × 10^5^ cells/mL was inoculated per well in 12-well plates. The cells were divided into three groups: (1) the normal control group: in which only serum-free culture medium was added; (2) the damaged group: in which serum-free culture medium containing 200 *μ*g/mL COM was added and incubated for 12 h; and (3) the protection group: CSPs with different molecular weights of 60 *μ*g/mL were mixed with 200 *μ*g/mL COM for 15 min and then cocultured with cells for 12 h. The cells were added diluted DCFH-DA and incubated for 30 min at 37°C. Then, the cells were washed 3 times with PBS. The fluorescence intensity was observed under a fluorescence microscope.
3. *Quantitative Analysis of the Proportion of Cells with Adhered Crystals*. 1.0 mL of cells suspension with a concentration of 1.0 × 10^5^ cells/mL was inoculated per well in 12-well plates. The cells were divided into two groups: (a) the COM damaged group: in which serum-free culture medium with 200 *μ*g/mL FITC-labeled nano-COM crystals was added and incubated for 12 h; (b) the protection group: CSPs with different molecular weights of 60 *μ*g/mL were mixed with 200 *μ*g/mL FITC-labeled nano-COM for 15 min and then cocultured with cells for 12 h. The 12-well plate was precooled for 30 min at 4°C, and then 200 *μ*g/mL FITC-labeled nano-COM crystals were added. The cells were cultured at 4°C for 1 h to inhibit cell endocytosis. After 12 h at 4°C, the cells were washed twice with cold PBS, PBS resuspended cells after pancreatin digestion, and the proportion of cells with adhered crystals was detected by flow cytometry.

### 2.7. Statistical Analysis

Experimental data were expressed by the mean ± standard deviation ($\bar{x}\pm \text{SD}$). The experimental results were analyzed statistically using SPSS 13.0 software. The differences of means between the experimental groups and the control group were analyzed by Tukey. If *P* < 0.05, there was significant difference; if *P* < 0.01, the difference was extremely significant; if *P* > 0.05, there was no significant difference.

## 3. Results

### 3.1. Degradation of Corn Silk Polysaccharide and Determination of -COOH Content

After degradation of 124 kDa corn silk polysaccharide CSP0 at different times with reference to the conditions in Table 1, five degraded polysaccharides, namely, CSP1, CSP2, CSP3, CSP4, and CSP5, with molecular weights of 26.1, 12.2, 6.0, 3.5, and 2.0 kDa, were obtained, respectively. With prolonged ultrasonic time, the molecular weight of the polysaccharide decreased, the solubility of the polysaccharide increased, and the viscosity decreased. These behaviors can enhance the biological activity of polysaccharide [20].

The content of -COOH in the six CSPs was determined by conductometric titration and converted into uronic acid content. After degradation, the uronic acid content of polysaccharide (25.6% to 31.3%) was higher than that of CSP0 (19.6%), and CSP3 with molecular weight of 6.0 kDa was the highest (31.3%); when the molecular weight was lower than or higher than 6.0 kDa, the uronic acid content decreased. The uronic acid content of CSPs is basically consistent with the results reported in the literature [15, 31].

### 3.2. FT-IR Characterization of CSPs

**Figure 1:**
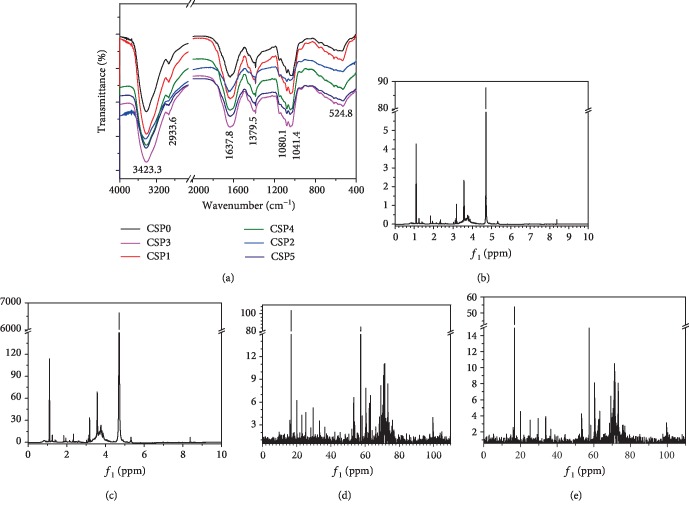
Structure characterization of CSPs. (a) FT-IR spectra of CSPs with different molecular weights. (b, c) ^1^H NMR spectra of CSP0 and CSP3. (d, e) ^13^C NMR spectra of CSP0 and CSP3.

Figure 1(a) shows the FT-IR spectra of CSPs with different molecular weights. The wide absorption band at 3,423.3 cm^−1^ corresponds to the absorption peak of the stretching vibration of –OH in the polysaccharide. The absorption band at 2,933.6 cm^−1^ corresponds to the stretching vibration of C–H. The vibration absorption peaks at 1,637.8 cm^−1^ were C=O and C=C. The peak at 1,379.5 cm^−1^ was a C–O stretching peak. The absorption peaks at 1,080.1 and 1,041.4 cm^−1^ indicated C–O–H stretching vibration in –COOH and C–O–C stretching vibration of ether bond in pyran ring [17, 32]. The peak at 1,080.1 cm^−1^ corresponds to galactan, and the peak at 1,041.4 cm^−1^ indicated arabinan side chains [32].

### 3.3. ^1^H and ^13^C NMR Characterizations of Corn Silk Polysaccharides

As shown in Figures 1(b) and 1(c), no significant change can be observed in the ^1^H NMR spectrum of CSP3 after degradation and CSP0 before degradation, indicating that ultrasonic degradation did not destroy the structure of the polysaccharide.

In the ^1^H NMR spectra of CSP0 and CSP3 (Figures 1(b) and 1(c)), the signal peaks appearing in *δ*3.16–5.31 ppm are glycosidic bonds of CSPs. The signal peak at *δ*2.35 ppm is CH_3_ proton of acetyl ester. The signal peaks at *δ*1.25 ppm and 1.08 ppm come from CH_3_ protons of deoxysugar. Methyl ester stroma was observed at 3.74 ppm [32].

*δ* 5.16, 4.24, 4.09, 3.81, 3.57, and 3.75 ppm belong to the chemical shifts of H-1 to H-6 of -4,6)-*α*-D-Glcp-(1; however, *δ* 5.16, 4.09, 4.03, 3.59, 3.55, and 1.11 ppm belong to the chemical shifts of H-1 to H-5 and CH_3_ of 3)-*α*-L-Araf-(1; *δ* 5.26, 3.75, 3.55, 3.59, 3.75, and 1.11 ppm belong to the chemical shifts of H-1 to H-5 and CH_3_ of *α*-L-Rhap-(1; *δ* 5.09, 3.81, 3.56, 3.81, 3.55, and 3.59 ppm belong to the chemical shifts of H-1 to H-6 of 4)-*β*-D-Galp-(1; *δ* 5.00, 3.55, 3.59, 3.81, 3.75, and 3.18 ppm belong to the chemical shifts of H-1 and H-6 of 3,5)-*β*-D-Manp-(1; *δ* 4.70, 3.59, 3.81, 4.24, and 4.09 ppm belong to the chemical shifts of H-1 and H-5 of *β*-D-Xylp-(1 [15]. The incomplete agreement with the hydrogen chemical shift on the standard monosaccharide may be due to the complex structure of the polysaccharides, and the interaction between groups changes the chemical shift of the hydrogen atom.

In the ^13^C NMR spectra of CSP0 and CSP3 (Figures 1(d) and 1(e)), the signal in the region of *δ* 100–104 ppm indicates that the monosaccharide in the polysaccharide exists in the form of a pyran ring. The signal peak at *δ* 42.25 ppm is designated as acetyl, and the signal at *δ* 53.26 ppm is designated as methyl carbon and methoxy group of the ester group [16]. As shown in Figure 1(d), *δ* 100.40, 79.43, 78.04, 75.94, 69.24, and 60.45 ppm belong to C1 to C6 signal peaks of -4,6)-*α*-D-Glcp-(1, respectively; *δ* 109.01, 81.74, 83.80, 70.25, 71.17, and 20.03 ppm correspond to the C1 to C5 and CH_3_ signal peaks of polysaccharide 3)-*α*-L-Araf-(1; however, *δ* 92.21, 73.37, 71.17, 71.91, 68.47, and 16.77 ppm belong to the C-1 to C-5 and CH_3_ signal peaks of *α*-L-Rhap-(1; *δ* 107.48, 76.83, 73.37, 75.62, and 69.24 and 60.45 ppm belong to the C-1 to C-6 signal peaks of 4)-*β*-D-Galp-(1; *δ* 98.98, 71.52, 75.94, 68.47, 73.37, and 62.41 ppm belong to the C-1 and C-6 signal peaks of 3,5)-*β*-D-Manp-(1; *δ* 107.48, 74.12, 76.83, 70.25, and 64.09 ppm are attributed to the chemical shifts of C-1 and C-5 on *β*-D-Xylp-(1 [15].

Based on the FT-IR, ^1^H, and ^13^C NMR detection results, the CSPs in the present study primarily consist of *α*-D-Glcp, *α*-L-Araf, *α*-L-Rhap, *β*-D-Galp, *β*-D-Manp, and *β*-D-Xylp, a result that is similar to that reported in literature [16, 33].

### 3.4. Antioxidant Capacity of Corn Silk Polysaccharides with Different Molecular Weights

**Figure 2:**
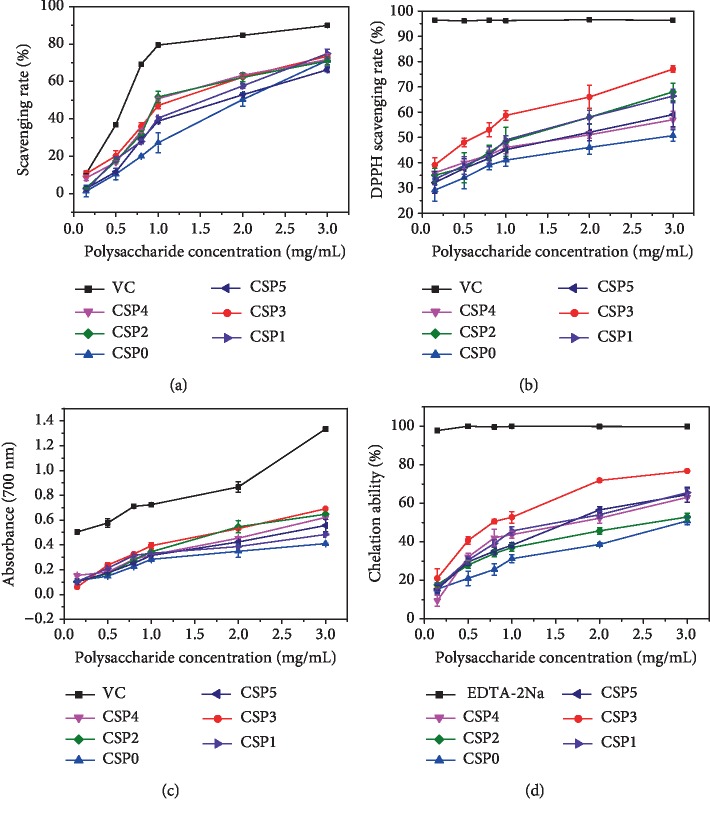
Antioxidant activities of CSPs with different molecular weights. (a) Hydroxyl radical scavenging capacity. (b) DPPH radical scavenging capacity. (c) Reducing power. (d) Chelating Fe^2+^ ion capacity.

1. *Scavenging Capacity of ·OH Radicals*. The production of ·OH may lead to cell senescence and tissue damage [34]; thus, ·OH scavenging is an important characteristic of antioxidant defense mechanism. The ·OH scavenging capacity of the six CSPs is shown in Figure 2(a). The ·OH scavenging ability of three polysaccharides with intermediate molecular weights (namely, CSP2, CSP3, and CSP4) was greater than that of the polysaccharides with larger (namely, CSP0 and CSP1) and smaller molecular weights (namely, CSP5).
2. *DPPH Radical Scavenging Capacity*. DPPH primarily exists in the form of nitrogen-containing free radicals and can accept single electron or hydrogen atom [25]. Therefore, the stability of DPPH radical is an important indicator to evaluate biological activity.  Figure 2(b) shows that CSP3 with a molecular weight of 6.0 kDa had the best DPPH radical scavenging ability. When the molecular weight of CSP was higher or lower than 6.0 kDa, the DPPH radical scavenging ability was reduced, and the crude polysaccharide CSP0 was the weakest.
3. *Reducing Power*. The reducing ability of CSP2, CSP3, and CSP4 with intermediate molecular weights was stronger than that of the other polysaccharides (Figure 2(c)). The weakest reducing power was observed in CSP0 with the largest molecular weight.
4. *Chelating Effect on Ferrous Ions*. Ferrous ions can cause Fenton reaction and free radical reaction and produce substances harmful to the human body [35].  Figure 2(d) is a comparison of the ability of each CSP to chelate Fe^2+^. Among the CSPs, CSP3 had the strongest while CSP0 had the weakest chelating ability.

According to the four indexes in Figure 2, although the rules of polysaccharides are not completely consistent, CSP2, CSP3, and CSP4 had the strongest antioxidant capacity, whereas CSP0 and CSP5 had the weakest antioxidant capacity. Polysaccharides with intermediate molecular weights, especially CSP3, had the strongest antioxidant capacity, whereas CSP5 with the lowest molecular weight and CSP0 with the largest molecular weight were weaker. The antioxidant activity of polysaccharides increased gradually with the increase in polysaccharide concentration, showing concentration-dependent behavior.

### 3.5. Regulation of Corn Silk Polysaccharides on Calcium Oxalate Crystal Formation

**Figure 3:**
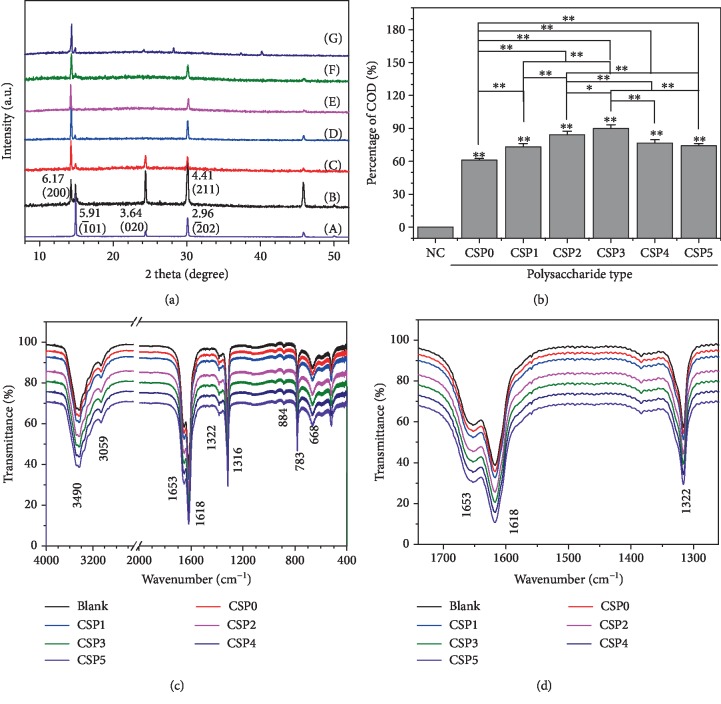
Characterization of CaOx formed in the presence of 0.5 mg/mL of CSPs. (a) XRD patterns: (A) blank control, (B) CSP0, (C) CSP1, (D) CSP2, (E) CSP3, (F) CSP4, (G) CSP5. (b) Percentage of COD in CaOx. (c, d) FT-IR spectra.

The effects of six CSPs on the formation of CaOx crystals, the main component of kidney stones, were compared.  Figure 3(a) is the XRD patterns of CaOx crystal formed in the presence of 0.5 mg/mL CSPs. In the absence of polysaccharides, only diffraction peaks attributed to COM appeared, and the diffraction peaks at 2*θ* = 14.88°, 24.2°, 30.16°, and 38.24° belong to the ($\overset{¯}{1}01$), (020), ($\overset{¯}{2}02$), and (130) planes of the COM crystal, respectively [36]. The addition of CSP can induce the formation of COD, and the diffraction peaks at 2*θ* = 14.28°, 30.12°, 32.28°, and 40.28° belong to the (200), (211), (411), and (213) planes of the COD crystal, respectively [37].

Quantitative calculations showed that the percentage of COD (73.1% to 84.1%) in the crystal induced by CSP initially increased and then decreased slightly (Figure 3(b)) as the molecular weight of CSP decreased; CSP3 with moderate molecular weight induced the highest percentage of COD (89.8%), whereas CSP0 induced the lowest percentage of COD (61.0%).

FT-IR detection consistently supported the aforementioned results (Figure 3(c)). In the absence of CSP, asymmetrical stretching *υ*_as_(COO^−^) and symmetrical stretching *υ*_s_(COO^−^) of CaOx appeared at 1,618 and 1,316 cm^−1^, respectively. At 3,490 to 3,059.2 cm^−1^, five stretching vibration peaks of O–H bond appeared in the crystal water, indicating that the CaOx formed was pure COM crystal [36].

After CSP was added, *υ*_as_(COO–) and *υ*_s_(COO–) in the CaOx crystals all showed blue shift in different degrees as the molecular weight of CSPs decreased from 124 to 2.0 kDa (Table 2). Varying blue shifts of *υ*_as_(COO–) and *υ*_s_(COO–) appeared at 1,618 to 1,653 cm^−1^ and 1,316 to 1,324 cm^−1^, respectively. This observation showed that the percentage of COM gradually decreased while COD gradually increased. Given that *ν*_as_(COO–) of COM and COD occur at 1,618 and 1,653 cm^−1^, respectively, *ν*_s_(COO–) of COM and COD were 1,316 and 1,324 cm^−1^, respectively (Figure 3(d)) [37, 38]. The blue shift values of *ν*_as_(COO–) and *ν*_s_(COO–) depended on the percentage of COD crystals in the COM–COD mixture.

### 3.6. Effect of CSP0 Concentration on Calcium Oxalate Crystal Formation

**Figure 4:**
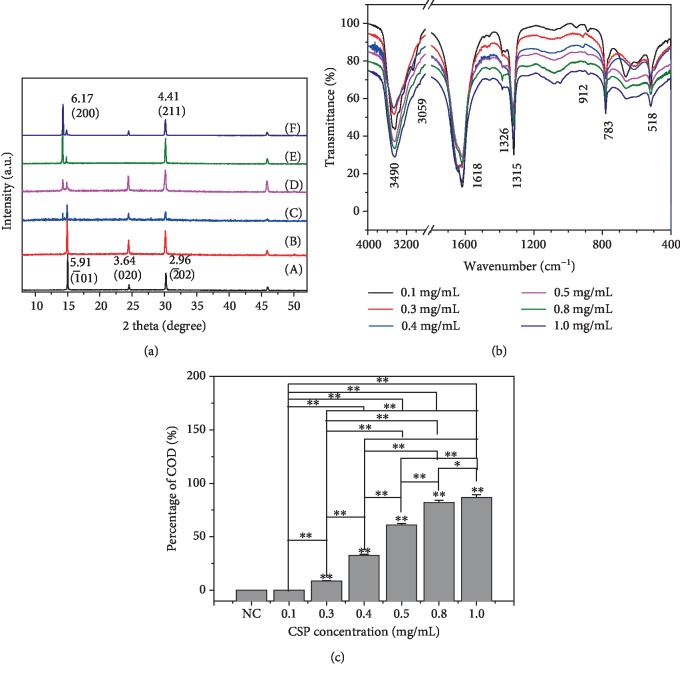
Characterization of CaOx crystals formed in the presence of different concentrations of CSP0. (a) XRD patterns: (A) 0.1, (B) 0.3, (C) 0.4, (D) 0.5, (E) 0.8, (F) 1.0 mg/mL. (b) FT-IR spectra. (c) Percentage of COD in formed CaOx.

Increased polysaccharide concentration also increased its ability to induce COD formation.  Figure 4(a) shows that the diffraction peaks of COD were strengthened as CSP0 concentration increased, suggesting an enhancement in the induced amount of COD. According to the quantitative calculation of *K* value method [9], in the presence of CSP0 of 0.1, 0.3, 0.4, 0.5, 0.8, and 1.0 mg/mL, the percentage content of COD in the generated CaOx crystal was 0, 8.6%, 32.6%, 61.0%, 82.0%, and 86.5%, respectively (Figure 4(c)).

Figure 4(b) and Table 3 show the FT-IR spectra of CaOx crystal as CSP0 concentration increased, *υ*_as_(COO–) blue shift continuously. In the fingerprint region, the absorption bands of COD crystals were at 912 and 668 cm^−1^, which were obviously different from 951, 887, and 518 cm^−1^ of COM, of which 887 and 783 cm^−1^ belonged to the COM C–C stretching vibration and O–C–O in-plane bending vibration, respectively [38].

### 3.7. Scanning Electron Microscopy Observation of Calcium Oxalate Crystals in the Presence of Corn Silk Polysaccharides

**Figure 5:**
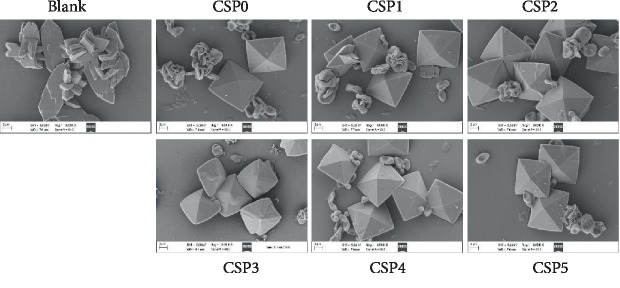
SEM images of CaOx crystals formed in the presence of 0.5 mg/mL CSPi. c(Ca^2+^) = c(Ox^2−^) = 0.60 mmol/L, c(NaCl) = 10 mmol/L; crystal growth time: 3 d. Magnification: 3000x.

Figure 5 shows the scanning electron microscopy (SEM) observations of CaOx crystals formed in the presence of each CSPi. In the absence of polysaccharides, most of the crystals formed were hexagonal COM crystals and aggregation. After adding 0.5 mg/mL CSPi, tetragonal bipyramid COD crystals appeared. The percentage of COD increased first and then decreased slightly as the molecular weight of CSP decreased. SEM observation results consistently support the corresponding XRD and FT-IR results.

In addition to the crystal phase change of CaOx, the crystal morphology also changed. The edges and corners of COM crystals induced by polysaccharide became round and blunt, especially CSP3, and the induced crystal size was relatively small.

### 3.8. Effects of Corn Silk Polysaccharides on Zeta Potential of Crystal

The zeta potential is a measurement of electrostatic repulsive force between particles. When the crystal surface has a high charge density, the absolute value of zeta potential is larger, the crystals do not aggregate easily, and the stability in the solution increases [38].

**Figure 6:**
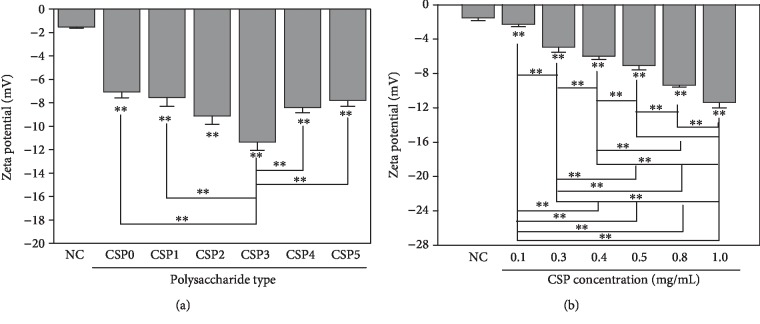
Zeta potential of CaOx crystals formed under CSP control. (a) Effects of molecular weight. (b) Effects of CSP0 concentration.

Figure 6 shows the zeta potentials of the formed CaOx crystal induced by CSPs.

1. For polysaccharides with different molecular weights, the zeta potential became more negative from -1.53 to -11.35 mV as the molecular weight decreased from 124 to 6.0 kDa. However, with further decreased molecular weight to 2.0 kDa, the zeta potential increased from -11.35 to -7.8 mV. The absolute value of zeta potential of the surface of CaOx crystal induced by CSP3 with moderate molecular weight was the largest
2. For one type of polysaccharide (such as CSP0), the zeta potential decreased from -1.53 to -11.39 mV as CSP0 concentration increased from 0 to 1.0 mg/mL. The more negative the zeta potential was, the more the negatively charged CSP molecules adsorbed on the crystal surface

### 3.9. TGA Analysis of Calcium Oxalate Crystals Formed in the Presence of CSPi with Different Molecular Weights

**Figure 7:**
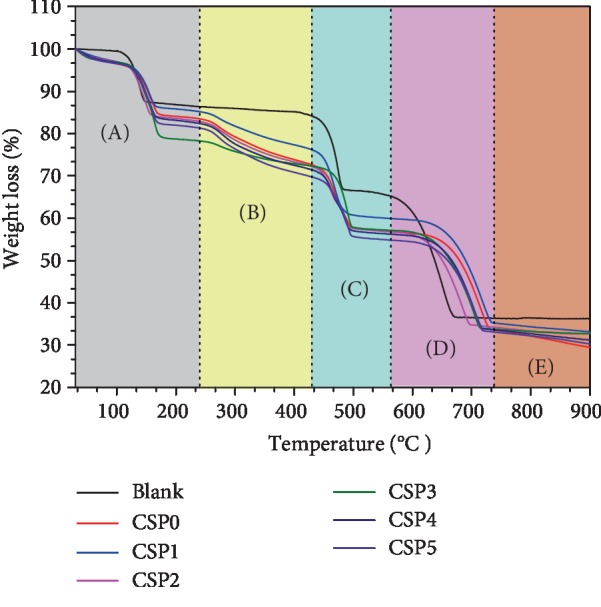
TGA analysis of CaOx crystals formed in the presence of 0.5 mg/mL CSPi with different molecular weights.

As shown in Figure 7, decomposition of CaOx crystals obtained in the blank group without polysaccharide was divided into three steps. The weight percentages lost were 12.18% (section A, 95.73°C, corresponding to the loss of structure and surface crystal water), 18.33% (section C, 409.14°C, corresponding to the decomposition of anhydrous crystals into calcium carbonate), and 28.99% (section D, 552.94°C, corresponding to decomposition into calcium oxide) (Table 4) [39, 40]. The theoretical weight loss of pure COM decomposed into CaC_2_O_4_, CaCO_3_, and CaO was 12.33%, 19.17%, and 30.12%, respectively, which is basically consistent with our results.

The TGA curve of CaOx crystals formed after adding 0.5 mg/mL polysaccharide was different from that of the blank group, which was attributed to the COD crystal induced by polysaccharide and CSPi adsorbed by crystal. The coordination bound water of polysaccharide-induced CaOx crystal was decomposition in the range of 25°C to 128°C (section A). As the temperature continued to rise to 200°C to 400°C, the polysaccharide molecules adsorbed on the crystal underwent thermal decomposition reaction (section B) [41–43]. As temperature reached 734°C, the CaOx sample was decomposed completely (section E in Figure 7). Finally, the residual weight percentages of CaOx crystals induced by CSP0, CSP1, CSP2, CSP3, CSP4, and CSP5 were 33.52%, 35.15%, 34.7%, 33.87%, 33.68%, and 33.19%, respectively.

No thermogravimetric loss occurred in the blank group at 200°C to 400°C (section B in Figure 7). However, the crystals induced by CSPs underwent thermal decomposition reaction at this temperature stage. Therefore, the weight loss at this stage can be considered as polysaccharide loss [41–43], that is, the weight of polysaccharide incorporated into the crystal [41]. As can be seen from Figure 7(B), the proportion of CSP3 incorporated into the crystal (4.93%) was the smallest, but its decomposition temperature was the highest (241.62°C). In contrast, the proportions of CSP0, CSP1, CSP2, CSP4, and CSP5 polysaccharides incorporated into the crystals were 9.72%, 7.67%, 9.24%, 9.78%, and 10.44%, respectively. However, their decomposition temperatures (221.19°C, 229.44°C, 226.74°C, 225.88°C, and 223.22°C) were lower than 241.62°C of CSP3. This result indicated that CSP3 had stronger specific interaction with crystals and stronger binding. Given that the incorporation of CSPi made the starting deposition temperature of the “CSPi-crystal” sample higher than that of the pure COM sample, the stability of the “CSPi-crystal” during heating was better than that of the pure crystal in the absence of polysaccharide [41].

### 3.10. Effects of CSPi on Soluble Ca^2+^ in Reaction System and Calcium Oxalate Precipitates

**Figure 8:**
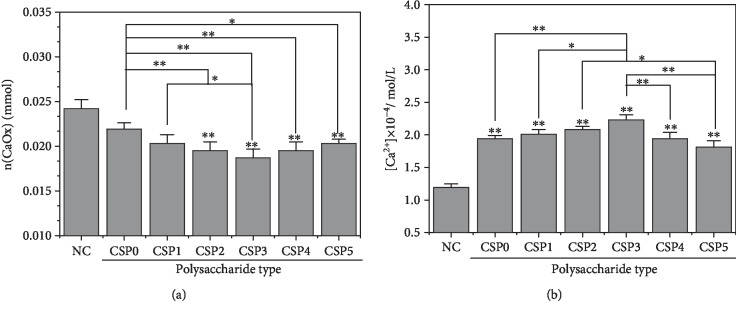
The molar amount of CaOx precipitation (a) and soluble Ca^2+^ concentration in the supernatant (b) in the presence of 0.5 mg/mL CSPs. c(Ca^2+^) = c(Ox^2−^) = 0.60 mmol/L, c(NaCl) = 10 mmol/L; crystal growth time: 3 d.

The mass of CaOx precipitate and the concentration of soluble Ca^2+^ in the supernatant measured by ICP after adding 0.5 mg/mL CSPi are shown in Figure 8. The molar amount of CaOx precipitate in the presence of CSPi was lower than that of the blank group (Figure 8(a)), and it was the lowest (0.0187 mM) in the presence of CSP3. After separation of CaOx precipitates, the concentration of soluble Ca^2+^ ions (0.181 to 0.223 mmoL/L) in the supernatant containing polysaccharide was higher than that in the blank group (0.119 mmoL/L) (Figure 8(b)), and it was the highest (0.223 mmoL/L) in the presence of CSP3, showing that CSP3 had the strongest ability to complex Ca^2+^. Figure 8 shows that CSP3 complexed the most Ca^2+^, so the concentration of soluble Ca^2+^ in the supernatant was the largest, whereas the amount of precipitate formed was the smallest, and CSP3 inhibited the formation of CaOx crystals to the greatest extent.

To verify the reliability of the results in Figure 8, we calculated the sum of the molar amount of soluble Ca^2+^ in the supernatant and the molar amount of Ca^2+^ in the CaOx precipitates (Table 5). The total molar amount of Ca^2+^ ions in each group was approximately 0.030 mmol, which was the same as the total molar amount of calcium in the reactant (0.030 mmol).

### 3.11. Effect of Polysaccharides on Crystallization Kinetics of Calcium Oxalate Crystals

**Figure 9:**
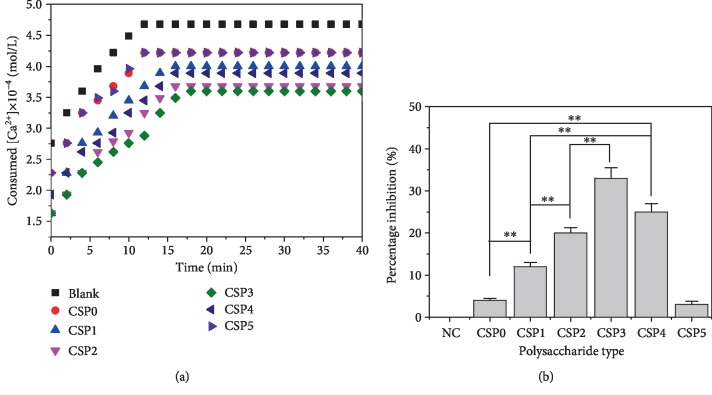
Consumed Ca^2+^ concentration in CaOx solution in the presence of 0.5 mg/mL CSPs (a). Inhibition percentage of CaOx crystallization by CSPi (b).

Figure 9 shows the effect of six CSPs on the crystallization kinetics of CaOx crystal. CSP can inhibit the crystallization process of CaOx. In 0.6 mmoL/L CaOx solution, the inhibition percentages of 0.5 mg/mL CSP0, CSP1, CSP2, CSP 3, CSP4, and CSP5 to CaOx crystallization were 4.1%, 12.5%, 20.6%, 33.1%, 25.5%, and 3.7%, respectively. The order of inhibition ability of CSPs to CaOx crystal crystallization was CSP3 > CSP4 > CSP2 > CSP1 > CSP0 > CSP5, and the inhibition effect of CSP3 with moderate molecular weight was the best, indicating that the addition of polysaccharides can obviously inhibit the crystallization of CaOx crystal.

### 3.12. Toxicity Assessment of CSPi on HK-2 Cells

**Figure 10:**
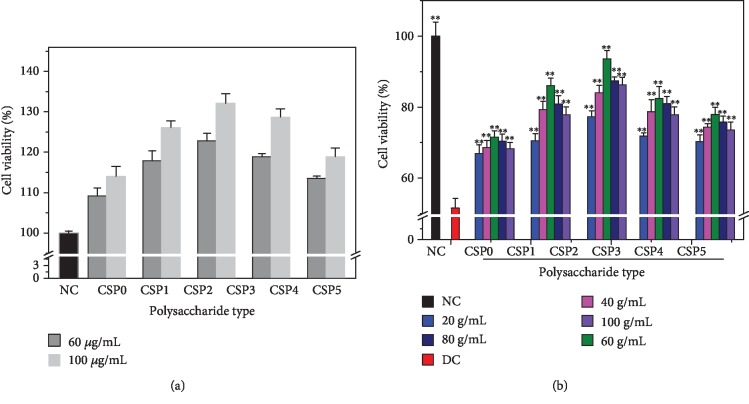
Cytotoxicity of CSPs with different molecular weights (a). Effect of COM crystals on the viability of HK-2 cells before and after CSP protection (b). NC: normal control; DC: damage control. COM concentration: 200 *μ*g/mL. Treatment time: 12 h. Compared with the DC group, ^∗^*P* < 0.05; ^∗∗^*P* < 0.01.

The CCK-8 method was performed to detect the toxicity of CSPi with different molecular weights on normal human kidney proximal tubular epithelial cells (HK-2) (Figure 10(a)). After 24 h of interaction between HK-2 cells and each CSPi, the cell viability was still above 100%. These results showed that these CSPi caused no cytotoxicity on HK-2 cells and promoted cell growth.

### 3.13. CSPi Protects HK-2 Cells from Calcium Oxalate Crystal Damage

Figure 10(b) shows the effect of COM crystals with a size of 100 nm before and after CSPi protection with different molecular weights on the viability of HK-2 cells. After damage of COM crystals for 12 h, cell viability decreased from 100% to 51.59%, showing that COM had obvious damage to HK-2 cells.

However, under the protection of CSPi with different molecular weights, the damage of nano-COM to cells was significantly reduced. The cell viabilities of the CSP1, CSP2, CSP3, CSP4, and CSP5 protection groups were 71.56%, 86.07%, 93.58%, 82.42%, and 77.91%, respectively, and CSP3 had the best protection effect.

### 3.14. CSPi Protection Reduces Reactive Oxygen Species Levels

Intracellular reactive oxygen species (ROS) increases when HK-2 cells are damaged. Excessive ROS can quickly react with intracellular macromolecules, causing damage to normal cell function and eventually cell death. This oxidative cell damage can promote the deposition of CaOx crystals in the kidney [44].

**Figure 11:**
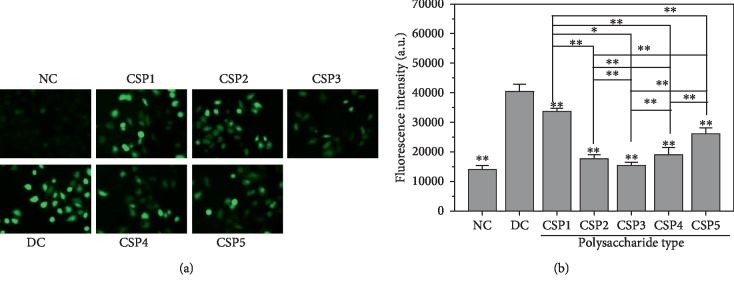
Effects of nano-COM crystals on ROS levels of HK-2 cells before and after CSP protection with different molecular weights. (a) The images of ROS distribution observed under fluorescence microscope. (b) Quantitative histogram. NC: normal control; DC: damaged control. Polysaccharide concentration: 60 *μ*g/mL. COM crystal concentration: 200 *μ*g/mL. Treatment time: 12 h. Magnification: 400x. Compared with the DC group, ^∗^*P* < 0.05; ^∗∗^*P* < 0.01.

Figure 11 shows the effect of nano-COM crystals on the ROS levels of HK-2 cells before and after CSPi treatment. ROS fluorescence intensity of the normal group was the lowest, that is, ROS was less. ROS fluorescence significantly increased after damage. ROS fluorescence intensity of the protection group was between those of the normal and injury groups, indicating that CSPi can protect cells from COM crystal damage.

### 3.15. Protection of Corn Silk Polysaccharides with Different Molecular Weights Reduces Adhesion of Crystals onto Cells

**Figure 12:**
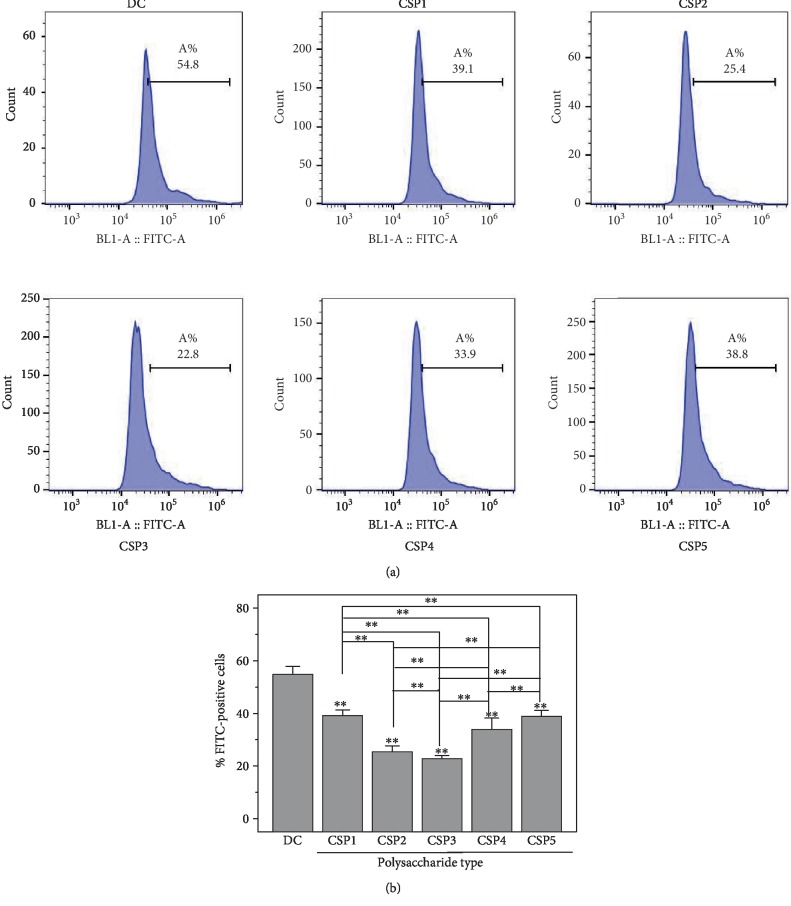
The proportion of cells with adhered COM crystals before and after CSP protection with different molecular weights. (a) Flow histogram. (b) Statistical results. DC: damaged control. Polysaccharide concentration: 60 *μ*g/mL. COM crystal concentration: 200 *μ*g/mL. Treatment time: 12 h. Compared with the DC group, ^∗^*P* < 0.05; ^∗∗^*P* < 0.01.

Figure 12 shows the proportion of cells with adhered COM crystals before and after CSP protection by flow cytometry. The proportion of cells in the injury group (54.8%) was significantly higher than that in the polysaccharide protection group (22.8% to 39.1%). In other words, CSP3 treatment had the strongest inhibition to the adhesion ability (22.8%) of COM crystals to cells.

## 4. Discussion

### 4.1. Causes of the Greatest Antioxidant Activity of CSP3 with Moderate Molecular Weight

(1) *Effect of Polysaccharide Molecular Weight*. As shown in Figure 2, CSP3 with a moderate molecular weight had the strongest antioxidant capacity, whereas CSP0 with the highest molecular weight and CSP5 with the smallest molecular weight had relatively low antioxidant capacity because polysaccharides with too large molecular weights have relatively compact structure, large molecular volume, large steric hindrance, and relatively few exposed active groups [45]. Ultrasonic degradation destroys the hydrogen bond between the intramolecular and intermolecular polymeric chains of the polysaccharide, making the molecular chain of the polysaccharide more expanded [46] and providing sufficient space to bind to the receptor, thereby improving its antioxidant activity.
 However, when the molecular weight of polysaccharide is too small, that is, the molecular volume is too small, the polysaccharide cannot easily form a spiral structure with biological activity, the chain structure is destroyed, and the molecular structure is loose [47]; hence, its activity is reduced.
 Xu et al. [48] ultrafiltrated and purified four kinds of molecular weight polysaccharides COP1 (7.9 kDa), COP2 (36 kDa), COP3 (83 kDa), and COP4 (225 kDa) from camellia seed polysaccharide. The authors found that COP2 and COP3 with moderate molecular weights had stronger free radical scavenging ability and reducing ability than COP1 and COP4. Im et al. [49] isolated three kinds of aloe polysaccharides with molecular weight from aloe and also found that polysaccharides with intermediate molecular weight (5 kDa < Mw < 400 kDa) had the strongest immunoregulation and antitumor activity on mouse macrophages. Shao et al. [50] found that *Sargassum* polysaccharide with the highest sulfate content and medium molecular weight had the strongest free radical scavenging ability, reducing ability, and antitumor activity.
(2) *Effect of Carboxyl (–COOH) Content in Polysaccharides*. The active group –COOH in polysaccharides can reduce the generation of ·OH and is positively correlated to the content of active groups [47, 51]. Given that CSP3 with a moderate molecular weight had the highest uronic acid content (31.3%, Table 1), that is, the highest content of –COOH, CSP3 had the strongest ability to provide H for ·OH and the strongest antioxidant activity. The reasons for the highest content of –COOH in CSP3 are as follows:
  - The shear force generated by cavitation at the initial stage of ultrasound destroys noncovalent bonds within and between polysaccharide molecules, polymer clusters will be depolymerized [52], glycosidic linkages are destroyed [20], and –COOH wrapped in molecules is exposed. Therefore, when the molecular weight is gradually degraded from CSP0 of 124 to 6 kDa (CSP3), the content of –COOH in the polysaccharides increases
  - However, with the extension of ultrasonic time, the mechanical force increases, the molecular weight of polysaccharide further decreases, and the volume of polysaccharide molecules further decreases. High-intensity ultrasonic energy may destroy some chemical bonds of the polysaccharides, resulting in decarboxylation reaction [53, 54]. Moreover, linear CSP molecules with galacturonic acid undergo different degrees of esterification, and the degree of esterification is high when the molecular volume is small [55]. Therefore, the content of –COOH in polysaccharides with low molecular weight (such as CSP4 and CSP5) decreases again

### 4.2. Reasons for CSPs to Inhibit Calcium Oxalate Monohydrate-Induced Calcium Oxalate Dihydrate Formation

**Figure 13:**
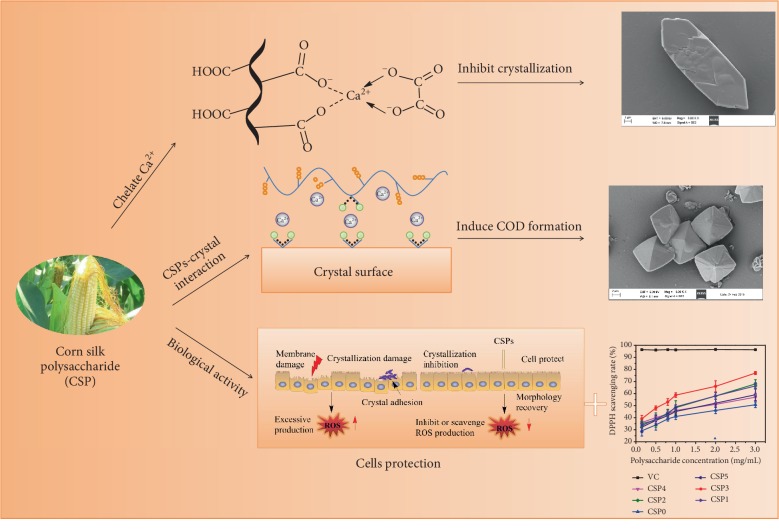
Schematic diagram of the regulation of CaOx crystals and protection effect on oxidative damage in renal epithelial cells in the presence of CSPs.

The schematic diagram of the regulation of CaOx crystals by CSPs with different molecular weights is shown in Figure 13. In the absence of polysaccharides, the generated CaOx crystals were all COM (Figures 3(a) and 5(a)). After adding CSPs, COD crystals were induced, and approximately 4.93% of CSP3, 7.67% of CSP1, 9.24% of CSP2, 9.72% of CSP0, 9.78% of CSP4, and 10.44% of CSP5 were incorporated into the crystals (Figure 7), forming a “CSPi-crystal” complex in which “CSP3-crystal” had the highest thermal decomposition temperature, that is, the best thermal stability. Given that the –COOH group in CSP can interact with the surface of COM crystal through calcium bridge (_(CSP)_ COO–... Ca^2+^ ... –OOC _(COM)_), which prevents free Ca^2+^ from penetrating into the lattice of COM, thus inhibiting the formation of COM crystal [28].

The –COOH groups in CSP can complex free Ca^2+^ in solution, increase Ca^2+^ enrichment on polysaccharide surface, and form high-energy interface on the polysaccharide surface. Moreover, the adsorption of Ca^2+^ by –COOH in CSP reduces the degree of freedom of Ca^2+^, increases the [Ca^2+^]/[Ox^2-^] ratio in local areas, and increases the energy state of calcium. Both high-energy interface and high-calcium energy state can promote thermodynamic metastable COD formation [56].

Given that CSP3 had a high content of –COOH, a small molecular volume, and the strongest complexing ability to free Ca^2+^ in solution, CSP3 had the best inhibitory effect on CaOx crystals and the highest percentage of induced COD (Figure 3(c)). COD is less toxic to renal epithelial cells than COM [57, 58], and COD is easier to excrete through urine [59]; therefore, inhibiting the formation of COM is more beneficial for inhibiting the formation of kidney stones.

### 4.3. Corn Silk Polysaccharides Induce Calcium Oxalate Monohydrate Crystals with Rounded Corners and Small Size

The corners and edges of COM crystals induced by polysaccharide became round and blunt, especially CSP3, and the induced crystal size was relatively small (Figure 5) because Ca^2+^ at the tip and edge of COM crystals easily coordinated with –COOH in the polysaccharides, resulting in dissociation-precipitation equilibrium of COM crystals. Continuous dissociation-precipitation eventually made the COM crystals round and blunt.

Calcium oxalate crystals with sharp edges and corners are more likely to cause cell membrane rupture, thereby exacerbating cell damage [60]. CSPs induced the formation of round blunt crystals, which reduced the possible mechanical damage of the crystals to the cells and reduced the generation of cell membrane debris, thus inhibiting the nucleation of microcrystals. In addition, small round blunt crystals are easier to be excreted out of the body with urine, this reducing the risk of crystal retention. Therefore, inducing the formation of small round blunt crystals is conducive to inhibiting the formation of stones.

### 4.4. CSPs Protect HK-2 Cells from Calcium Oxalate Crystal Damage

The schematic diagram of the protection effect on oxidative damage in renal epithelial cells in the presence of CSPs is shown in Figure 13. CSPs with different molecular weights not only did not express toxicity to HK-2 cells but also promoted growth (Figure 9(a)). The protection of CSPs with different molecular weights on HK-2 cells inhibited the damage of COM crystal to cells, enhanced cell viability (Figure 9(b)), and reduced the level of ROS (Figure 10). Oxidative stress induced by ROS and free radical damages cell structure and function of biomolecules, leading to the formation of many diseases [47]. CSPs can effectively scavenge free radicals (Figure 2), reduce the level of ROS, protect cells from damage, and inhibit the adhesion of crystals onto cells (Figure 11). CSPs' inhibition of COM crystal adhesion is also related to its ability to increase the negative charge on the surface of COM crystal after adsorption on the crystal surface, thereby increasing the absolute value of zeta potential (Figure 6). High absolute value of zeta potential can increase the repulsive force between crystal and cell and reduce the adhesion between them [8, 61]. The protection ability of each CSPi is consistent with its antioxidant ability, that is, CSP3 had the greatest cell protective ability.

Li et al. [62] reported that *Lycium barbarum* polysaccharide (LBP-4a) has protective effects on KBrO_3_-induced renal injury in rats. Protection of LBP-4a can increase the activity of antioxidant enzymes in rat kidney tissue, decrease mitochondrial membrane potential, and reduce DNA damage in renal cells. Li et al. [63] reported that laminarin has a protective effect on glycerol-induced kidney injury in mice, and laminarin with moderate molecular weight has the best protective effect on the kidney. Acidic polysaccharides can enhance antioxidant enzyme level and reduce oxygen free radicals.

## 5. Conclusions

CSP0 with a molecular weight of 124 kDa was degraded by ultrasonication, and five degraded polysaccharides with molecular weights of 26.1, 12.2, 6.0, 3.5, and 2.0 kDa were obtained. The structure was characterized by FT-IR and NMR analyses. These CSPs exhibited scavenging activity on hydroxyl and DPPH, as well as reducing power and chelation ability for ferrous ions. Each CSPi can increase the concentration of soluble Ca^2+^ in the solution, reduce the quality of CaOx crystals, inhibit COD to COM conversion, increase the absolute value of zeta potential of the crystal surface, and reduce the degree of crystal aggregation and COM crystal size. These CSPs induced no cytotoxicity on HK-2 cells, protected HK-2 cells from damage by nano-COM crystals and increased cell vitality, and reduced the level of ROS and the adhesion of crystals onto cells. The activity of CSPs was closely correlated with the molecular weight, i.e., very high or very low molecular weight of CSPs was not conducive to CSP activity. CSP3 with a moderate molecular weight had the best antioxidant effect and the best cell protection effect. Overall, our results suggested that each CSPi, especially CSP3, may be potential antistone drugs.

## Figures and Tables

**Table 1:** Degradation conditions and basic physicochemical properties of CSPs with different molecular weights.

CSPs	Ultrasonic degradation time^∗^ (min)	Intrinsic viscosity [*η*] (mL/g)	Mean molecular weights Mr (kDa)	Uronic acid content (%)
CSP0	0	9.35 ± 3.54	124	19.6
CSP1	15	4.55 ± 0.94	26.1	26.8
CSP2	17	3.19 ± 0.33	12.2	29.6
CSP3	20	2.29 ± 0.3	6.0	31.3
CSP4	27	1.67 ± 0.3	3.5	28.0
CSP5	30	1.3 ± 0.7	2.0	25.6

^∗^Ultrasonic power: 600 W; ultrasonic frequency: 40 kHz.

**Table 2:** FT-IR characteristic absorption peaks of CaOx crystals induced by CSPs with different molecular weights.

CSPs	*ν*_as_(COO^−^) (cm^−1^)	*ν*_as_(COO^−^) (cm^−1^)	*ν*_s_(COO^−^) (cm^−1^)	COM (cm^−1^)	COD (cm^−1^)	COM (cm^−1^)	COM (cm^−1^)	COD (cm^−1^)	COM (cm^−1^)
CSP0	1650.7	1618.0	1315.0	950		886	783 sh	669	515
CSP1	1652.2	1625.2	1322.3	947		885	783 sh	667	516
CSP2	1652.5	1625.2	1322.3	948	913	885	783	667	518
CSP3	1652.7	1631.1	1323.1	952	913	887	783	662	517
CSP4	1652.2	1631.8	1324.5	952	913		783 sh	669	518
CSP5	1651.8	1631.8	1323.1		912		783 sh	663	518
Pure COD	1653		1324		912		783	668	
Pure COM		1618	1316	951		887	783 sh		518

Note: sh: sharp.

**Table 3:** FT-IR characteristic absorption peaks of CaOx crystals induced by different concentrations of CSP0.

Concentrations of CSP0	*ν*_as_(COO^−^) (cm^−1^)	*ν*_as_(COO^−^) (cm^−1^)	*ν*_s_(COO^−^) (cm^−1^)	COM (cm^−1^)	COD (cm^−1^)	COM (cm^−1^)	COM (cm^−1^)	COD (cm^−1^)	COM (cm^−1^)
0.1	1642	1617.9	1315	951		888	783 s	665	515
0.3	1646	1618.2	1315	950	912		778 s		519
0.4	1648.5	1618.2	1315	950	914		783 s		517
0.5	1650.7	1618.6	1319	950		888	781 s	662	518
0.8	1652.5	1625.2	1323			883	781 sh	667	520
1	1653	1626	1325			886		668	519
Pure COD	1653		1328		912		783	668	
Pure COM		1618	1316	951		887	783 sh		518

Note: s: strong; sh: sharp.

**Table 4:** TGA analysis of CaOx crystals formed in the presence of CSPs with different molecular weights.

CSPs	A		B		C		D		Residual weight (%)
	Decomp. *T*^∗^ (°C)	Weight lost (%)	Decomp. *T* (°C)	Weight lost (%)	Decomp. *T* (°C)	Weight lost (%)	Decomp. *T* (°C)	Weight lost (%)	
Blank	95.73	12.18	—	—	409.14	18.33	552.94	28.99	36.55
CSP0	96.11	12.43	221.19	9.72	411.6	16.80	580.82	23.14	33.52
CSP1	99.45	11.02	229.44	7.67	406.35	16.72	584.59	15.59	35.15
CSP2	90.93	13.33	226.74	9.24	410.33	15.59	568.00	21.97	34.70
CSP3	102.21	18.12	241.62	4.93	416.20	15.08	573.40	23.19	33.87
CSP4	98.78	13.50	225.88	9.78	410.88	15.41	582.69	22.33	33.68
CSP5	100.14	14.40	223.22	10.44	417.48	14.99	583.19	21.41	33.19

Note: ^∗^Decomp. *T*: decomposition temperature.

**Table 5:** Effect of CSPs with different molecular weights on CaOx crystal phase, soluble Ca^2+^ concentration in solution, and CaOx precipitate mass.

CSPs	Uronic acid content (%)	COD (%)	m(Ca^2+^) (mg/L)	c(Ca^2+^) (mmol/L)	n(Ca^2+^) (mmol)	Mass of CaOx (g)	Molar amount of CaOx (mmol)	Total Ca^2+^ (mmol)
Blank	—	—	4.742	0.119	0.0060	0.0031	0.0242	0.0302
CSP0	19.6	61.0	7.740	0.194	0.0097	0.0027	0.0210	0.0307
CSP1	26.8	73.1	8.032	0.200	0.0100	0.0026	0.0203	0.0303
CSP2	29.6	84.1	8.306	0.208	0.0104	0.0025	0.0195	0.0299
CSP3	31.3	89.8	8.932	0.223	0.0110	0.0024	0.0187	0.0297
CSP4	28.0	76.6	7.753	0.194	0.0097	0.0025	0.0195	0.0292
CSP5	25.6	74.1	7.220	0.181	0.0091	0.0026	0.0203	0.0294

Notes: m(Ca^2+^): quality concentration; c(Ca^2+^): molar concentration; n(Ca^2+^): molar amount.

## Data Availability

The data used to support the findings of this study are available from the corresponding author upon request.
